# Narrative abilities in individuals with Down syndrome: single case-profiles

**DOI:** 10.3389/fpsyg.2023.1116567

**Published:** 2023-10-03

**Authors:** Isabel Neitzel

**Affiliations:** Research Unit of Language and Communication, Department of Rehabilitation Sciences, TU Dortmund University, Dortmund, Germany

**Keywords:** narration, Down syndrome, single cases, language and cognition, profile analysis

## Abstract

**Introduction:**

Narrative abilities are an important part of our everyday lives and social interaction with others. Nevertheless, narration is a complex ability influenced by language and cognition. This makes it difficult for individuals with language and cognitive impairment, such as in children and adolescents with Down syndrome. Previous studies have shown distinct narrative impairments in individuals with Down syndrome; nevertheless, this research was based on overall group means in most cases. To identify individual strengths and weaknesses and to draw conclusions for speech and language therapy, the narrative profile of every participant should be considered equally. Following this approach, the current study aims to describe single case narrative profiles in individuals with Down syndrome.

**Methods:**

The narrative transcripts of 28 children and adolescents with Down syndrome (aged 10;0–20;1), based on a non-verbal picture book, were rated using the Narrative Scoring Scheme across seven macro- and microstructural categories. Point scores across the whole group are displayed – nevertheless, the paper specifically addresses the individual narrative profiles of the participants. The participants could be assigned to narrative profile groups which show different characteristics, strengths and weaknesses. Group comparisons and correlations were computed for the relation to language abilities (especially vocabulary) and nonverbal cognitive abilities.

**Results:**

The results of the two profile groups with minimal and developing narrative skills differ significantly not only concerning narrative outcomes in the Narrative Scoring Scheme but also for language abilities and developmental stage of nonverbal cognition. Individuals that show floor effects in narrative abilities are characterized by an overall weakness in language and cognition. In contrast, a group of approximately equal size shows distinct strengths in their narrative profiles which are in line with their vocabulary strengths, MLU and nonverbal cognition.

**Discussion:**

The current study uses a new approach to identify individual narrative profiles in a group of individuals with Down syndrome. The results of the investigation underline the existence of narrative impairments in many individuals with Down syndrome but also point to individual strengths of the participants. Furthermore, the study outcomes suggest that narrative abilities might be representative for overall language and cognition in individuals with Down syndrome. However, intervention studies addressing narration are missing.

## 1. Introduction

Narration as a cultural technique plays an important role in social interaction. The social act of narration occurs in the exchange of two or more persons and linguistically comprises the text type narrative. According to Katz-Bernstein and Schröder (2017), this is defined as follows: “[Narratives contain] unique events that contain a special feature, in the form that something unexpected has happened. (...) Quite essential for narration in contrast to reporting is that with the unexpected, the breaking of the plan, an emotional evaluation accompanies it. An important function of narrative is to convey this emotion to the listener” (Katz-Bernstein and Schröder, 2017, p.2, translated by author). Narrative thus goes far beyond functional language and includes formal and communicative parts, as pointed out by Bamberg (2016). Previous investigations have shown a high impact of narrative competences on literacy acquisition (Botting, 2002; O’Neill et al., 2004) and school outcomes (e.g., O’Neill et al., 2004 for mathematical abilities). Regardless of its high relevance in everyday communication, narration is a distinctly complex skill, which is linked to cognitive and linguistic performances, e.g., the Theory of Mind (Tompkins et al., 2019) or the vocabulary of a narrator (Korecky-Kröll et al., 2019; Neitzel, under review). Narratives represent a distinct manifestation of the superordinate expressive form text (Kintsch, 1974; De Beaugrande and Dressler, 1981). In the narrative, successive sentences are linked in terms of content and language – only through this connection does a story emerge. The contextual connection of utterances, the so-called coherence, enables listeners to connect to their own prior and world knowledge (De Beaugrande and Dressler, 1981) and serves as content orientation within a narrative (Swoboda, 2011). This usually follows a conventionalized, recurring form, the so-called macrostructure. This macrostructure is a formal blueprint of a story, which according to Stein and Glenn’s (1979) story grammar model consists of six grammar units (setting, initiating event, internal response, attempt, outcome, response; detailed introduction in Govindarajan and Paradis, 2022). Within a story, there can be so-called story episodes, i.e., different plot lines or events, which are, however, each structured according to the story grammar model. The three grammar units initiating event, attempt and outcome are obligatory, while other elements can be variably located in their position (internal reaction of characters) or optionally added (morality; Van Dijk, 1980). Macrostructure is thus a formal structure that is realized across content. Impairments of the macrostructure, which can occur in the context of developmental language disorders, manifest themselves, for example, through missing grammar units or an unstructured narrative sequence. Microstructure, on the other hand, “refers to a local level of analysis” (Govindarajan & Paradis, 2022, p. 1363) and includes all concrete linguistic information, e.g., word choice or sentence length. The concrete linguistic realization of narrative content is implemented at the microstructural level through the use of cohesive devices. These include, for example, conjunctions – which connect individual sentences – or pronouns, which enable references across sentences. Overall, microstructure encompasses “measures of word frequency, proportion of content words (i.e., nouns and verbs), grammaticality and sentence complexity” (Altman et al., 2022, p. 1119). Impaired language abilities can manifest themselves on a microstructural level, for example, through morphological or syntactic errors or an insufficiently differentiated vocabulary.

The complex interplay of different language levels and cognitive abilities, as well as the demands of the narrative form itself, lead to limitations in narrative ability. These have been observed in different populations. Narrative abilities of speakers with Down syndrome – a group of individuals characterized by multiple cognitive and language disabilities – have already been investigated in the literature by various research groups. Due to the extent of previous findings and research designs, a complete literature review is not possible here; reference is made to, among others, an extensive review by Segal and Pesco (2015). However, the current state of research can be clustered based on the following assumptions and the respective methodological focus: A large part of the international research concludes that speakers with Down syndrome show strengths in the macrostructure of a narrative (Keller-Bell and Abbeduto, 2007; Finestack et al., 2012; Segal and Pesco, 2015). In parallel (and rarely overlapping methodologically), some studies focused on the (underlying) language impairments of narrators with Down syndrome, highlighting impairments primarily at the microstructural level (Finestack et al., 2012; Channell et al., 2015; Ashby et al., 2017). This is illustrated subsequently on the basis of selected studies.

A study by Finestack et al. (2012) illustrates a comprehensive assessment of the narrative performance of speakers with Down syndrome. It focused on both macro- and micro-structural levels, and included a comparison group of typically developing children aged 4–6 years. They surveyed the narrative ability of 24 English-speaking adolescents and young adults (chronological age: *M* = 16;11 years, *SD* 3;2 years, range 12;1–23;4 years; mental age: *M* = 4;11 years, *SD* = 1;0 years, range 3;4–7;1 years) using the Narrative Scoring Scheme (NSS, Heilmann et al., 2010; see section 2.2). In an individual matching of (non-verbal) mental age between participants with Down syndrome and the control group (*n* = 21), the speakers with Down syndrome showed a macrostructure appropriate for their non-verbal mental age and significantly outperformed the typically-developing participants in terms of the macrostructure element introduction as well as the total score. Similarly, Neitzel and Penke (2022a) were able to show in a profile comparison of children with typical development and participants with Down syndrome with a mean non-verbal mental age of 5;03 years (in y;mm) that the narrative performance of the participants with Down syndrome – measured by NSS-scores – corresponded to that of 5-year-old typically developing children on group average. Such findings contribute to the widespread assumption that narrators with Down syndrome might show relative confidence in macrostructure (Segal and Pesco, 2015) – as measured by non-verbal cognitive stage of development, not chronological age.

In contrast, the results on microstructural performance in people with Down syndrome are more equivocal, which may be partly due to the methodological approach. Many studies in the literature have used MLU as a microstructural measure of narrative ability. Nevertheless, there are mixed findings in the literature on MLU in narratives of participants with Down syndrome. MLU is repeatedly used as an overall measure of grammar in narrative studies (e.g., Ashby et al., 2017; Channell, 2020), with high MLU indicating higher grammar skills. Neitzel and Penke (2021), in contrast, were able to show that higher MLU in participants with Down syndrome may rather be a manifestation of syntactic impairment. In their study, the MLU of participants with Down syndrome was even slightly higher than the MLU of a 9-year-old comparison group of typically developing children, despite a mean mental age of 5;03 years. This finding, however, was not caused by a high syntactic complexity, but was an expression of long but syntactically incoherent sentences. Accordingly, MLU is an important covariate concerning the morpho-syntactic abilities of individuals with Down syndrome in research, but great caution must be exercised in interpreting the pure values. The assumption that a higher value automatically indicates higher grammatical abilities does not apply unreservedly to these participants. The extent to which looking only at the group mean of the MLU can be misleading in the interpretation of narrative performance, can be demonstrated by the study of Finestack et al. (2012), in which participants were compared with typically-developing children of the same non-verbal mental age. The group mean in MLU of the two groups was comparable (differences n.s.). Strengths of the participants with Down syndrome were evident in the macrostructure, with these participants even outperforming the typically-developing children in some cases. However, when participants from both groups were matched 1:1 according to their MLU, no differences in favor of the participants with Down syndrome were detectable anymore. Firstly, this indicates that the group mean in MLU led to a distorted picture of individual performance. Secondly, this points to a strong interaction between macro- and microstructure, which makes it methodically difficult to differentiate between both constructs. These critical points lead to concerns regarding the interpretation of narrative performance on group level in individuals with Down syndrome. Overall, the presented research overview provides a partly ambiguous picture of narrative abilities in persons with Down syndrome.

Group evaluations, especially the focus on group mean comparisons and significances, have their value for basic deduction in narrative research. At the same time, the existing research lacks a focus on the individual performance of the participants, although individual aims, e.g., for speech and language therapy, can only be chosen on a case-by-case basis. Inferences from group results to the individual case may leave individual strengths and weaknesses undiscovered. Particularly speaking about narrative performance, which encompasses a wide range of abilities, group means are not necessarily informative about what individual narrative support for children and adolescents with Down syndrome should look like. Therefore, the present focus on single cases is primarily intended to provide clinical and educational conclusions and to demonstrate an exemplary approach to making research on individuals with Down syndrome more individualized. The investigation presents an analysis of individual performance profiles with a focus on the question of whether definable subgroups and competence profiles emerge in this context. The assessment should produce an overarching narrative profile for each case, encompassing macro- and micro-structural aspects of narrative competence, both of which are essential to narrative. For this reason, an assessment tool (Narrative Scoring Scheme, *cf.* 2.2) is used which allows for an overall view per case (total score), but at the same time allows for the derivation of individual support approaches on a case-by-case basis (identification of resources and weaknesses). At the same time, however, group performances are presented, in order to classify the narrative abilities of the examined children and adolescents with Down syndrome against the background of past research results.

## 2. Methods

### 2.1. Participants

Twenty eight children and adolescents with Down syndrome participated in the current study (free trisomy: *n* = 26; mosaic trisomy: *n* = 1; type of trisomy unknown: *n* = 1). All participants were recruited from institutions such as special needs schools and inclusive sports clubs^1^ with the aid of parent associations. The participants with Down syndrome were monolingual German speakers (15 f., 13 m.) and attended an inclusive (*n* = 17) or special needs school (*n* = 11). All individuals were Caucasian. The educational level of the families was variable, but high overall (15 mothers and fathers each with university entrance qualifications and/or academic degrees). Sufficient ability in hearing and vision was reported for all individuals (unimpaired hearing in *n* = 19 participants, mild hearing loss 10–30 dB in *n* = 9 participants). Participant characteristics and outcomes from cognition and vocabulary measures (see section 2.3) as well as MLU (see section 2.2) are presented in Table 1. The sample consists of older children, adolescents, and few young adults with Down syndrome (chronological age: *M* = 14;05 years;months). In the cognition test SON-R the participants scored on average 59.93% of the points and in the expressive vocabulary test AWST-R (description of both tests in 2.3) 66.28% of the points. The MLU of the participants is high (*M* = 7.00), but this is due to syntactic impairments in many participants (Neitzel and Penke, 2021). The research project involving the data presented here was approved by the Ethics Committee of the Medical Department of the University of Cologne (number of approval 18–121). Inclusion criteria for participation were growing up monolingual and verbal utterance skills at least at two-word level.^2^ Section 2.4 describes the procedure administered in the study.

**Table 1:** Participant characteristics concerning age, cognition, vocabulary and MLU across the group.

Instrument	Chronological age (in y;mm)	SON-R 2 ½-7 (Cognition) Reasoning Scale Raw score (max. 46 p.)	AWST-R (Vocabulary) Raw score (max. 75 p.)	Frog Story MLU (in words)
Mean	14;05	27.57	49.71	7.00
*SD*	2;06	6.18	13.44	2.94
Range	10;00–20;01	17–42	12–64	1.57–13.28

### 2.2. Narrative measures

The present evaluation of narrative abilities was conducted on the basis of written transcriptions of the narratives that the participants produced using the so-called Frog Story. The Frog Story is a nonverbal picture book (‘Frog, where are you?’; Mayer, 2003), including 24 black and white illustrations, that is widely used in research on narrative abilities. The required noun vocabulary for the story (acting characters and central objects/locations) was secured in advance using picture naming in a prepared PowerPoint presentation.^3^ The Frog Story as well as the scoring procedure (see next paragraph) has already been used successfully with participants with Down syndrome by Finestack et al. (2012). The research procedure, which is described below, is internationally common in this form and goes back to a study by Reilly et al. (2004). This approach could be used congruently for the participants with Down syndrome. The Frog Story picture book was introduced by the experimenter (‘Look, I have this book for you. I want you to tell me a story about it in a moment. First, let us look at the book together. Just look at the pictures.’). The book was presented nonverbally by the experimenter, who slowly leafed through it in a way that was clearly visible to the child. For each illustration, the child was given sufficient time to look at it, but no linguistic request was made yet. Subsequently, the participant was asked to tell the story on the basis of the illustrations. To do this, the book was flipped through page by page again, with the experimenter using only non-specific questions such as ‘What is happening here?’

Written transcripts of the narratives were made using ELAN 5.3 (Max Planck Institute for Psycholinguistics, The Language Archive, 2018). Transcription was controlled by two additional, individual raters (trained student assistants). Disagreements were discussed and resolved through a consensus process. Intelligibility of the individuals was partly limited by phonologic errors but did not affect the narrative analyses.^4^ The participants’ narrative ability was evaluated using the Narrative Scoring Scheme (NSS; Heilmann et al., 2010) in seven subcategories with zero to five points (max. 35 points). The NSS allows for the assessment of narrative performance using predetermined categories and a point scoring system and is widely used in narrative research. For the Frog story, comprehensive scoring examples are available to identify an immature (1 point), developing (3 points), and mature (5 points) narrative performance per subcategory (Miller et al., 2003).^5^ This handout by Miller et al. (2003) has since been translated into German in an expanded form and is freely available (Neitzel and Meier, 2023). For each category, the handout indicates exactly for which narrative content and linguistic features which score is to be assigned. Neitzel and Meier’s (2023) manual was evaluated in a study on transcripts from 89 typically developing children. For example, this may look like the following for the “conclusion” category: For this category, three central ‘events’ have been named which characterize the content of the story’s ending (Miller et al., 2003) – (a) The boy and the dog find the frog, (b) The boy takes a baby frog as a pet, (c) The boy waves/says goodbye and is happy. The manual indicates exactly how many points may be awarded each time a certain number of events are mentioned, e.g., 0 points for no event, 1 point for one event, 3 points for 2 events, 4 points for 3 events. In addition, 2 points are awarded if 1–2 events are mentioned and the end of the story is abrupt but clear (e.g., by the phrase “And over.”). Five points are awarded if the narrative is completely rounded off, possibly by common (German) phrases such as “And if they did not die, they are still alive today.” Thus, the evaluation can be done very specifically by trained raters. An interrater review revealed a very good reliability of 0.93 (95% CI: 0.77–0.98; Meier and Neitzel, 2023). Note that Heilmann et al. (2010) originally described the NSS to be a macrostructural instrument (clear for subcategories introduction, mental/emotional states, conflict/resolution, cohesion, and conclusion assess macrostructural skills). Nevertheless, the categories of character development – where choice of words is really important – and referencing – which interferes with grammar abilities by scoring, e.g., sentence linking – are more associated with the microstructure of a story. In the current investigation, core microstructural measures are number of different verbs and MLU. Nevertheless, NSS-scores should be considered as a combination score evaluating macro- and microstructural abilities.

### 2.3. Further standardized measures

The children’s and adolescents’ cognitive abilities were assessed using the reasoning scale of the SON-R 2 ½-7 nonverbal intelligence test (*Snijders-Oomen Non-verbal intelligence test-revised*; Tellegen et al., 2007). This instrument includes three subtests with 46 items in total: categories, analogies, and situations, and allows participants to respond both verbally or nonverbally. The reported reliability for the reasoning scale is 0.83 (Tellegen et al., 2007). The test is normed for a developmental age of 2;6 to 7;11 years (years; months). The SON-R has been used successfully in many studies with participants with Down syndrome (including Witecy and Penke, 2016). Since the instruction and response of the children can be non-verbal, no adaptation of the implementation was necessary. With respect to their language comprehension abilities, all participants were able to understand the instructions for the measures used.^6^ The vocabulary abilities of participants with Down syndrome were assessed by applying the AWST-R (*Aktiver Wortschatztest für 3- bis 5-jährige Kinder*; Kiese-Himmel, 2005), a widely used German productive vocabulary test containing 75 items (51 nouns, 24 verbs), presented with increasing difficulty and normed for three- to five-year-old typically developing children. The internal consistency of the AWST-R is α = 0.88 (Kiese-Himmel, 2005). A possible adaptive approach to test administration and scoring for participants with Down syndrome is described in Neitzel et al. (2021). For the present sample, however, no adaptation was necessary compared to the manual-faithful implementation.

### 2.4. Procedure

The participants were tested as part of a research project on the narrative skills of people with Down syndrome at University of Cologne. The participants took part in three test sessions of 45 to 60 min each. Written parental consent was obtained beforehand. In addition, at the first appointment, parents and child were verbally informed and a parent questionnaires on developmental history was handed out. At the first appointment, in addition to contact games, the SON-R 2 ½–7 (non-verbal cognition) and the expressive vocabulary test AWST-R were administered. In the second session further tests, mainly morpho-syntactic, were administered which are not part of this paper. The frog story narratives were collected in test session three. Each of the sessions was interrupted by appropriate rest breaks. Most of the testing took place in the participant’s home environment and some in the institutional environment (school). However, for each participant the testing location was kept constant across all three sessions. Participants were given a small, age-appropriate gift (e.g., sweets and pens) as a thank you for their participation in the study. Parents were also given a detailed report of their child’s test performance to give to their child’s speech and language therapist or teacher.

### 2.5. Data analysis

Results for the standardized measures were calculated according to the manual. Only raw scores were used in the present analyses. MLU in words as overall grammar measure and number of different verbs as a measure of verb vocabulary were calculated on the basis of the written transcripts of the narratives. Data processing in the current study was conducted using *SPSS 28* (IBM Corp, 2021). Analyses included the following steps: First, each participant’s narrative profile was manually assessed by two independent raters using the NSS. Mean, *SD* and range were calculated for each NSS category and total score. According to this profile (individual scores in the NSS categories), the participants were manually assigned to profile groups, which differed according to the distinguished ability levels of the NSS: minimal ability (1 p. in mean), developing ability (3 p. in mean) and advanced ability (5 p. in mean). Pearson’s correlations were used to examine the relationship between NSS scores (total score) and performance on various language and cognitive measures. In addition, non-parametric group comparisons were computed with respect to the language and cognitive variables and narrative performance (Mann–Whitney *U*-tests). To allow for a better understanding of the case profiles, individual associations and dissociations between NSS scores and language or cognition measures were considered using median split analyses and exact Fisher tests in each participant. The total score for the sample is reported in the results section and compared to group studies from the literature in the discussion to allow for classification despite the single case focus.

## 3. Results

### 3.1. Narrative group overview and profile groups at single case-level

Following the objective of the present study to not only rely on group means when assessing the narrative abilities of children and adolescents with Down syndrome, but to focus more on individual performance, each individual with Down syndrome was assigned to a profile group according to her narrative abilities. In addition, the Supplementary material of this article provides a complete overview of the narrative performance (NSS subpoints per category) provided by each individual case. The categorization that is typically made in the NSS was selected as a more reliable criterion, namely the differentiation between an immature (1 point), developing (3 points), and mature (5 points) narrative performance. A profile group was therefore created for a participant’s mean score of 1, 3, and 5 points in narrative performance. This resulted in the following distribution: Profile group (1) including participants showing minimal narrative abilities with a point score per subcategory of 0 to 1.99 points (*M* = 1); group (2) including participants showing developing narrative abilities defined by a category score of 2.00 to 3.99 points (*M* = 3); group (3) including participants showing advanced narrative abilities with a category score of ≥ 4 points (*M* ≥ 4). Since a mean value of 5 points can hardly be achieved in purely mathematical terms due to a maximum 5 points per category, *M* ≥ 4 was set as the criterion for profile group (3). Sixteen individuals could be assigned to profile group (1) – minimal narration, MN – and 12 individuals met the criteria of profile group (2) – developing narration, DN – while no participant could be assigned to profile group (3). Table 2 shows the mean NSS-scores of the participants in each profile group in addition to the total group. The results consistently show a higher point score in the DN group than in the MN group in all individual categories and in the total score. According to the mean values, the introduction (*M* = 3.83) and the cohesion (*M* = 3.17) are strengths of the DN group. However, in line with the results of the MN group (*M* = 0.75), the participants in the DN group also achieved low scores (*M* = 1.25) in the subcategory ‘conflict/ resolution’. As no participant in the sample reached more than 2 points, this category was not included in the assignment to the profile groups. Regarding the present forms of trisomy (*n* = 26 participants with free trisomy, *n* = 1 each with unknown and mosaic trisomy), it can be reported that the participant with unknown form of trisomy was located in the profile group MN. The participant with mosaic trisomy showed a performance corresponding to the DN profile group.

**Table 2:** Mean NSS-scores (SD; range) for profile groups of individuals with Down syndrome concerning narrative abilities with 0–5 points (p.) per subcategory.

Mean (*SD*; Range)	Total group (*n* = 28)	Profile group (1): minimal narration (MN) (*n* = 16)	Profile group (2): developing narration (DN) (*n* = 12)	Profile group (3): advanced narration (*n* = 0)
Introduction (0–5 p.)	2.71 (1.41; 0–5)	1.88 (1.20; 0–4)	3.83 (0.72; 3–5)	--
Character Development (0–5 p.)	1.79 (1.13; 0–5)	1.19 (0.66; 0–2)	2.58 (1.16; 1–5)	--
Mental/emotional states (0–5 p.)	1.61 (0.92; 0–4)	1.13 (0.50; 0–2)	2.25 (0.96; 1–4)	--
Referencing (0–5 p.)	1.96 (1.14; 0–4)	1.25 (0.86; 0–3)	2.92 (0.67; 2–4)	--
Conflict/ solution (0–5 p.)	0.96 (0.58; 0–2)	0.75 (0.58; 0–2)	1.25 (0.45; 1–2)	--
Cohesion (0–5 p.)	2.25 (1.08; 0–4)	1.26 (0.81; 0–3)	3.17 (0.55; 2–3)	--
Conclusion (0–5 p.)	1.79 (0.96; 0–4)	1.38 (0.62; 0–2)	2.33 (1.07; 1–4)	--
NSS total score (max. 35 p.)	13.07 (5.90; 1–26)	9.13 (3.67; 1–13)	18.33 (3.77; 14–26)	--

No participant in the sample reached more than 2 points in the subcategory ‘conflict/resolution.’ Therefore, this category was left out of the assignment to the profile groups.

**Figure 1:**
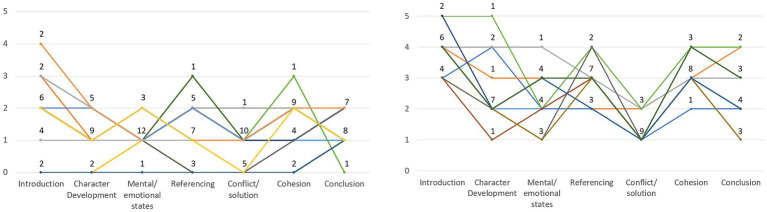
Overview of participants in narrative groups minimal narration (MN, left) and developing narration (DN, right). Each line represents one individual participant. The data labels indicate in each case how many participants have reached this value. For participants with an overlapping curve section, only a single line is displayed for visual reasons. Full narrative profiles per case are displayed in Supplementary Table S1.

The number of participants in the respective profile group indicates that a comparable number of individuals show a minimal and developing narrative performance whereas no participant can be categorized as a strong narrator. Figure 1 contrasts the individual narrative profiles of all participants from the two groups (please see full narrative profiles per case in Supplementary Table S1). It is clear that in group DN, there are significantly more downward deflections (individual weaknesses) than upward deflections (strengths). A few participants from group MN also show individual scores that exceed three points, but this is always only a single category per participant that exceeds the two-point mark.

### 3.2. Relations to cognition and language abilities

The presented narrative profiles show differing competencies concerning narrative abilities in children and adolescents with Down syndrome. The question remained open whether the individual assignment to these profile groups might be reflected by cognition or language abilities of the individuals. The relation between these factors was investigated in the subsequent analyses.

In this context, correlation analyses were first used to investigate which characteristics and abilities of the participants are associated with narrative abilities. Included here were the children and adolescents’ chronological age, cognition outcomes (SON-R raw scores) and MLU as overall grammar measure, as these have been consistently associated with narrative abilities in individuals with Down syndrome in the research literature (e.g., Finestack et al., 2012; Hogan-Brown et al., 2013; Channell et al., 2015). Based on recent findings that narrative abilities in individuals with Down syndrome are highly associated with vocabulary performance (Neitzel and Penke, 2022b) and specifically verb vocabulary (number of different verbs; see analyses in Neitzel, under review), these two expressive vocabulary measures were included. Table 3 shows the results of these analyses, which display significant correlations (*p* < 0.05/*p* < 0.001) between NSS-total scores and all measures except from chronological age.

**Table 3:** Correlations of narrative abilities (NSS-score) and age, cognition or language variables for all participants (n = 28).

	Total NSS-score	Chronological age	Raw score cognition	Raw score expr. vocabulary	*n* different verbs in Frog story	MLU in words
Total NSS-score		0.262	0.706**	0.841**	0.736**	0.721**
Chronological age			0.658**	0.299	0.137	0.154
Raw score cognition				0.681**	0.437*	0.584**
Raw score expr. vocabulary					0.627**	0.614**
*n* different verbs in Frog story						0.578**
MLU in words						

Difference is significant for *p < 0.05, **p < 0.001.

Since the correlation results did not provide any insight whether individuals in the two profile groups – profile group (3) (advanced performance) was disregarded at this point, as no participant met the criteria for it – differed in terms of their performance on the variables included (see Table 4), the performance was analyzed per profile group. The results indicate that the narrative ability profiles indeed reflect performance in other language and cognition measures. Accordingly, a non-parametric group comparison (Mann Whitney U) revealed a significant difference for cognition raw scores, vocabulary measures and MLU between the two profile groups (each *p* < 0.001). The number of different verbs used in the narratives was also significantly higher among participants in the DN group (*M* = 34.83) than in the MN group (*M* = 23.31; *p* = 0.006). At the same time, the examined participants of the two profile groups do not differ with regard to their chronological age and thus regarding their language experience.

**Table 4:** Mean results (SD, range) concerning age, cognition and language variables across profile groups and results of non-parametric group comparison (Mann–Whitney *U*).

Mean (*SD*; Range)	Total NSS-score (max. 35 p.)	Chronological age (in y;mm)	Raw score cognition (SON R 2 ½-7) (max. 46 p.)	Raw score expr. Vocabulary (AWST-R) (max. 75 p.)	*n* different verbs in Frog story	MLU in words
Profile group minimal narration (MN, *n* = 16)	9.13 (3.67; 1–13)	13;7 (2;4; 10;00–18;8)	24.06 (4.43; 17–31)	49.71 (13–67; 12–58)	23.31 (10.23; 4–37)	5.49 (2.67; 1.57–10.83)
Profile group developing narration (DN, *n* = 12)	18.33 (3.77; 14–26)	15;6 (2;7; 11;0–20;01)	32.25 (5.01; 25–42)	58.83 (5.46; 45–64)	34.83 (7.28; 25–48)	9.02 (1.83; 6.7–13.2)
Group comparison (*p*-values)	<0.001*	0.066	<0.001*	<0.001*	0.006*	0.001*

*Difference is significant for *p* < 0.05.

**Figure 2:**
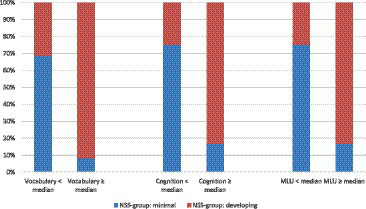
Individual associations and dissociations between language/cognition measures and narrative performance (NSS-score groups minimal narration (MN) and developing narration (DN)) (median splits).

To investigate the narrative performance of participants in the two profile groups more deeply regarding their cognitive and language abilities, a median-split analysis was conducted to compare associations and dissociations between sub-median performance and above median performance on each measure and the assignment to group MN or DN. The data for this comparison is visualized in Figure 2. Concerning their performance in the standardized expressive vocabulary measure AWST-R, 16 participants reached scores < median (< 54,00 points) and 12 participants showed scores ≥ median. Remarkably, the number is equal for the cognition measure (SON-R, median score 27.5) and MLU (median score 7.14). Given that 16 participants were assigned to group MN following their NSS-score and 12 participants to group DN, their narrative performance seems to be reflected very closely by sub-median or above median performance in the cognition and language measures. Concerning associations and dissociations, a comparable image is displayed for every measure: whereas 22 participants display associations between a lower or higher performance in the cognition/ language measure and their narrative ability (e.g., vocabulary < median & group MN or vocabulary ≥ median & group DN), 6 participants display a dissociation between these measures. 5 participants were assigned to group DN although they show vocabulary skills < median, while one participant was assigned to group MN and showed vocabulary skills ≥ median. In the cognition measure and MLU, 4 participants display sub-median performance and were still assigned to group DN, whereas two participants show above median performance but low narrative skills (group MN). Exact Fisher tests for all comparisons are significant (vocabulary: *p* = 0.002, cognition & MLU each: *p* = 0.006).

## 4. Discussion

The present study investigated narrative skills based on stories collected from 28 children and adolescents with Down syndrome using a non-verbal picture book. In comparison to previous studies, this study did not focus on group mean analyses, but assessed each child’s individual narrative profile. The point scores, that are differentiated in the NSS instrument, have been used as an orientation for the formation of narrative ability profile groups, which ranged from minimal to developing abilities and showed similarities with the children’s and adolescents’ performance in other language and cognitive domains. The detailed results of the present study are discussed below.

### 4.1. Narrative performance in individuals with Down syndrome

To allow for a general understanding of the data of the current investigation, the narrative abilities of individuals with Down syndrome are briefly discussed on group level (overview in Table 2). Results are classified with regard to previous studies, especially the NSS-scores described by Finestack et al. (2012). Afterwards, the data are discussed on a single-case level (with regard to the defined profile groups).

#### 4.1.1. Group level

The group results indicate that children and adolescents with Down syndrome show a significant limitation in the area of storytelling skills. Although the Frog Story could be performed with all participants, there was a wide point range in the categories of the NSS. This underlines a considerable heterogeneity concerning performance in the narrative task. While some participants were able to convey very little narrative content and – in some cases – scored zero in several categories, there were also 42.9% of individuals (*n* = 12) who displayed a developing narrative performance with an average of three points in the NSS. However, the majority of participants (*n* = 16, 57.1%) scored less than two points per category on average. In accordance with the scoring guidelines of the NSS, this points to an immature or minimal narrative ability in the early stages of development. Neitzel and Penke (2022a) were able to show that, despite these limitations, the performance of the current sample (in mean) corresponded to the performance of 5-year-old typically developing children and was, thus, appropriate for their calculated mental age of 5;03 years on average. At the same time, the results showed a higher range than those of typically developing children, which is one reason why this paper aims at a more in-depth, case-by-case analysis of narrative skills (see 4.1.2). Methodically, it is important to underline in this context that the comparison of individuals with Down syndrome and typically-developing children must be handled very carefully and is often criticized, which also includes the use of (non-verbal) mental age for matching-procedures. In the current analyses, no mental age was therefore used as a benchmark for narrative or other developmental levels. Nevertheless, previous analyses such as the findings from Neitzel and Penke (2022a) involved this method and are therefore reported accordingly.

While the categories introduction and cohesion represented a relative strength of the participants, the children and adolescents with Down syndrome only achieved an extremely low mean score of 0.96 points (range 0–2 points) in the category conflict/resolution. This suggests that individuals with Down syndrome are often unable to realize the central conflicts and resolutions of a narrative linguistically, which can significantly impede listeners’ understanding of the story. While the finding that subjects with Down syndrome scored relatively high in the introduction and cohesion categories is consistent with the findings of Finestack et al. (2012), the participants in the current study showed a distinctly lower score in the conflict/resolution category than was the case with Finestack et al. (2012) (group mean 13.07 vs. 17.21). This may suggest that the distinct impairment in the important narrative feature of realizing conflict and resolution, that the participants showed here, cannot be generalized for all individuals with Down syndrome. It is also possible that the differences could have arisen during implementation, for example, if the Finestack et al. (2012) study had provided assistances that were not detailed in the paper. Alternatively, the differences could have occurred during the scoring process, as the NSS recommendations by Heilmann et al. (2010) only provide guideline scores for items 1, 3, and 5, which are orientational in nature. The point values 2 and 4 could therefore have been assigned according to different criteria, likewise the point value 0. The discussed scores on the (macrostructural) NSS subcatgories introduction, cohesion, and ‘conflict/resolution’ indicate both strengths and weaknesses regarding the macrostructure of a story in the studied participants with Down syndrome. At first glance, this seems to contradict the assumption of other authors that individuals with Down syndrome often show strengths at the macrostructural level (e.g., Keller-Bell and Abbeduto, 2007; Finestack et al., 2012; Segal and Pesco, 2015). However, since the category ‘conflict/resolution’ might be a difficulty for typically developed children as well, as examined by Neitzel and Penke (2022a), it also seems possible here that this category represents a particular complexity for storytellers in the learning process. Therefore, this category may need to be further explored in future studies in order to distinguish possible syndrome-specific difficulties of people with Down syndrome from an (expected) delay in narrative skills. The fact that this study cannot specifically address macro- and microstructure due to its focus on an overarching narrative profile of people with Down syndrome is, however, a limitation of this paper.

The remaining ambiguities as well as the great standard deviations in the category results indicate that group comparisons are only of limited use to represent the narrative performance of individuals with Down syndrome. Moreover, they do not allow for individual derivations of clinical implications. The following discussion of individual cases is intended to make this possible.

#### 4.1.2. Single case-level

As described, each participant exhibits a different and individual narrative profile in the current investigation. At the same time, certain profile groups seem to emerge, which allow a rough orientation concerning the narrative performance level; these profile groups are remarkably reflected in the linguistic abilities and the non-verbal developmental level, measured by SON-R raw scores. A large proportion of participants (*n* = 12, MN group) showed minimal narrative abilities, which were accompanied by limitations in vocabulary (weaker performance as measured by the other group) and lower MLU. Since MLU is often distorted in participants with Down syndrome, in this case it cannot be assumed *per se* that the individuals produced shorter utterances than the other participants with Down syndrome (see detailed discussion of the relation to linguistic and cognitive factors in section 4.2). However, there is a significant difference from the group with developing narrative abilities (DN) in terms of performance in the nonverbal cognition measure. A lower developmental level in nonverbal cognition would be a possible explanation for the occurring floor effects in group MN. However, because of the overall relatively low language performance (as measured by vocabulary measures and MLU), an alternative hypothesis would be that these participants show the most severe limitations in their linguistic-cognitive profile among the participants of the study, and that the low narrative abilities might be only one of several impairments. It is a limitation of the present study that no comparison data from other populations with intellectual disabilities was obtained that would allow a more precise interpretation of the results.

The present results provide a first insight that the striking heterogeneity in the narrative performances of participants with Down syndrome – as measured by group means in previous studies, e.g., by Finestack et al. (2012) – could possibly be explained by different narrative profiles and developmental stages. In this context, it would also be helpful to conduct intervention studies that could help to examine the skills of individual groups. On the other hand, these could also be used to implement an even stronger focus on the individual case, since the profile groups presented here naturally also represent a form of clustering.

The analyses presented here shed light on narrative performance and possible profiles of individuals with Down syndrome and thus not only open up further research areas, but also point to clinical implications. Even if the presented results are only a rough orientation and explorative in nature (see also limitations in section 4.3), the different narrative profiles indicate different developmental levels in storytelling skills. Since narration is an important basic ability of interaction in our everyday life, work on the narrative level should not be left out in participants with Down syndrome. It is necessary to examine in the form of further investigations – above all in intervention studies – which concrete starting points result from the individual performances.

### 4.2. Associations of narrative abilities and language or cognition variables

In the last step, this study investigated whether the cognitive and language impairments of individuals with Down syndrome are related to their narrative abilities. Previous research suggests that narrative abilities in individuals with Down syndrome might be related to cognition, vocabulary performance and MLU. These measures were therefore considered as possible factors influencing narrative performance in speakers with Down syndrome in this context. The correlation analyses performed show a clear correlation between narrative performance, measured by the NSS score, cognition and all language measures. An exception is chronological age, which shows no significant correlation. In this regard, the results underline that chronological age might be no determining factor for language performance in individuals with Down syndrome (evidence of exceptions exists, e.g., for grammar comprehension, see Witecy et al., 2021). Furthermore, the results point to the many factors and skills involved in a (successful) narrative. The individual linguistic and cognitive skills are nevertheless interrelated according to the correlation analyses, which makes a clear picture of directional connections difficult. Moreover, with regard to the individual case-oriented evaluation, the correlation analyses do not provide any insight.

The individual cognitive and linguistic measures were therefore considered separately for the different defined profile groups. Non-parametric group comparisons reveal significant differences in all cognitive and language measures except chronological age. The two profile groups are thus comparable in their mean chronological age, but independently show completely different language and cognition profiles. For all measures considered, a clear difference in favor of individuals in the DN group concerning the achieved values can be observed, which indicates a better linguistic performance and a higher stage of cognitive development in the respective participants. The difference is very pronounced for MLU, but due to the morpho-syntactic impairments in many individuals with Down syndrome, the interpretation of this difference should be made with caution; thus, since the MLU of the participants is high overall across the individual profile groups, this does not necessarily indicate lower performance in the MN group. Rather, it must be remembered that a higher MLU of the participants in many cases occurs due to syntactic deficits, for example, sentence entanglements. Neitzel and Penke (2021) were able to show in a syntactic analysis for the sample presented here that the participants showed a high degree of sentence fragmentation. These are evidence of the syntactic deficits present in many participants. Likewise, the analyses showed the described sentence entanglements, for example in the following sentence: *“Wir wissen noch nicht was sind die beiden was meint.”* ‘We do not know yet what the two are meaning’ (Neitzel & Penke, 2021, p. 8).

A median split analysis was performed to relate individual associations and dissociations between the measures expressive vocabulary, cognition - measured by SON-R raw score - and MLU to the results of the profile group comparisons. In the individual assignments (*cf.* Figure 2) one can see that individuals with abilities < median are more likely to be assigned to the MN group, whereas individuals that show abilities ≥median are more likely to be assigned to the DN group. Despite repeated indications in previous analyses on the current sample that the vocabulary of the participants might be decisive for narrative abilities (see Neitzel and Penke, 2022b; Neitzel, under review), none of the three included variables could be identified as salient in the median split analyses and exact Fisher tests.

The described profiles point to implications for clinical work with the respective participants. On the one hand, it shows that different individuals with Down syndrome can reach a very variable level of (narrative) performance in adolescence. The participants in group DN might have an even higher narrative potential, which could be supported by addressing individual communicative strengths – nevertheless, since no longitudinal data is available in this investigation, this cannot be verified. This also has important clinical implications for the MN group, who may appear to have low levels of language or narrative skills, as AAC methods could be used more intensively in speech and language therapy with these participants rather than focusing solely on spoken language. In this context, it is a limitation of the present research that for the narrative analysis, only spoken language was included in the transcripts. For the future, it would be desirable to focus also on the non-verbal communication of the participants, for example pragmatic skills or gestural communication, and to investigate whether possible gesture usage might add supplementary narrative content to the children’s and adolescents’ output.

### 4.3. Limitations

The current study provides novel insights into single case-profiles of individuals with Down syndrome with regard to their narrative abilities, which may be transferable to other domains of language and cognition. At the same time, however, the study also underlies some limitations.

The described profile groups reflect the individual narrative abilities of children and adolescents with Down syndrome. Since corresponding comparison profiles of individuals from other clinical populations – e.g. individuals with (mixed) other intellectual disabilities – are not available, no statement can be made at this point as to whether these profiles are syndrome-specific for Down syndrome. A generalizability of the results is therefore not given, however, due to the selected individual case-oriented approach also not necessarily the goal of the study. The profile classifications shown are intended as a suggestion for the case-by-case classification of narrative performance and do not represent fixed categories.

Although the investigated sample of 28 children and adolescents with Down syndrome is of good size compared with other studies involving this population, the number of participants <30 individuals represents a statistical limitation. The formation of profile groups from this sample results in small numbers of participants representing each narrative profile (< 20 persons). It would be desirable to conduct similar analyses with a large number of participants, ideally >60 persons, to investigate whether tenable and statistically distinguishable profile groups can be verified for a larger group of individuals. At last, the sample studied shows little overall variability in terms of socioeconomic status and ethnicity. This should be taken into account in the recruitment of future samples. Another methodological limitation is that the MLU was calculated using the narrative transcripts presented here. Due to the already proven use of this material with participants with Down syndrome, a significant over- or underestimation of the utterance length is not to be assumed, but this cannot be completely excluded.

## 5. Conclusion and future research

The present study provides insights into the narrative abilities of children and adolescents with Down syndrome. The novel approach used here was to characterize individual narrative profiles, created on the basis of the NSS, beyond the performance of the whole group. It was found that the defined profile groups differed not only in terms of their narrative ability, but also in their general linguistic-cognitive profile. This allows the conclusion that narrative abilities could possibly be considered representative of the further linguistic-cognitive performance profile; however, further research in this area is necessary to draw firm conclusions. In this regard, the present analyses should only represent a starting point to conduct further investigations of individual narrative profiles and to explore individual developmental potentials, especially in the context of intervention studies. The aim of the present study was to provide a narrative profile per case, but not to examine individual aspects of macro- and microstructure in participants with Down syndrome in depth. However, as the data of the present article are fully available in open access, it is an intention of the author to initiate further research.

## Data availability statement

The raw data supporting the conclusions of this article will be made available by the authors, without undue reservation. The full transcripts of the participants are available at CHILDES database: https://childes.talkbank.org/access/Frogs/German-Neitzel.html.

## Ethics statement

The studies involving humans were approved by Ethics Committee of the Medical Faculty at University of Cologne. The studies were conducted in accordance with the local legislation and institutional requirements. Written informed consent for participation in this study was provided by the participants’ legal guardians/next of kin.

## Author contributions

IN collected the data, developed the analytical concept for the project, including the analyses presented in this manuscript, computed the all results, and wrote the manuscript.

## Footnotes

^1^Inclusive sport clubs are a leisure activity that can be attended independently of schooling in Germany. In this case, it is an association where parents can have their children with Down syndrome cared for on weekends as part of preventive care.

^2^Mental age, as calculated using SON R 2 ½-7 (see section 2.3) > 3;6 years was another inclusion criterion. Raw scores from the SON-R are presented in Table 1, whereas mental age is not part of the current analyses.

^3^The occurring nouns were presented to the participants as separate picture details and were to be named by the children. If a child was unsure about an item or named it incorrectly, the experimenter named the word correctly and spoke briefly with the child about the respective item. At the end of the review, this noun was then asked again. The children were able to name the core vocabulary of the respective stories in all cases at least in the second run. Afterwards, the children began to look at the original book.

^4^Full narrative transcripts of all participants with Down syndrome are available at CHILDES database: https://childes.talkbank.org/access/Frogs/German-Neitzel.html.

^5^The intermediate values 2 and 4 points were defined within the project and applied comparably by all raters. The interrater reliability for the evaluation of the transcripts, which was carried out on the basis of this manual, could be estimated as very high in a study by Meier and Neitzel (2023) [intraclass correlation on the basis of the two-factorial unadjusted random model, 0.93 (95%-CI: 0.77–0.98)].

^6^Instruction on the measures used was given using simple SVO sentences (word order in German: subject, verb, object). Instructional comprehension was secured as part of the overarching research project by use of a standardized grammar comprehension measure (German version of the TROG). All children passed the first comprehension blocks in which simple sentence structures are tested.
