# Supplementary material for: Narrative abilities in individuals with Down syndrome: single case-profiles
Source: Front Psychol. 2023 Oct 3;14:1116567. doi: 10.3389/fpsyg.2023.1116567 (PMC10579590; doi:10.3389/fpsyg.2023.1116567)
Supplement: Supplementary file 1 [file Table_1.pdf]

Supplementary material

Supplementary Table 1

Values per subcategory for each participant in the NSS coding. Data are sorted chronologically within the Minimal Narration (MN) and Developing Narration (DN, shaded cells) groups

ID	Introduction	Character Development	Mental/ emotional states	Referencing	Conflict/ solution	Cohesion	Conclusion	Group
3	1	1	2	1	1	1	2	MN
4	3	1	1	1	1	2	2	MN
6	3	2	1	2	2	2	1	MN
7	0	0	1	0	0	0	1	MN
8	1	1	2	1	1	1	1	MN
9	4	2	1	2	1	3	0	MN
10	2	1	1	1	1	1	2	MN
14	2	1	1	2	1	2	2	MN
16	1	1	1	0	0	1	2	MN
19	2	1	1	2	1	2	1	MN
20	0	0	0	0	0	0	1	MN
24	2	2	1	3	1	2	1	MN
26	2	2	1	2	1	2	2	MN
27	4	2	1	1	1	2	2	MN
28	1	1	1	1	0	2	1	MN
29	2	1	2	1	0	2	1	MN
2	3	4	2	2	1	2	2	DN
11	4	3	3	2	2	3	4	DN

12	4	4	4	3	2	3	3	DN
13	3	2	1	3	1	3	1	DN
15	4	2	2	3	1	3	1	DN
17	5	5	2	4	2	4	4	DN
18	5	2	3	3	1	4	3	DN
21	3	1	2	3	1	3	2	DN
22	3	2	1	4	1	3	2	DN
23	4	2	1	3	1	3	1	DN
25	4	2	3	2	1	3	2	DN
30	4	2	3	3	1	4	3	DN
